# MLH1 Deficiency Down-Regulates TLR4 Expression in Sporadic Colorectal Cancer

**DOI:** 10.3389/fmolb.2021.624873

**Published:** 2021-05-07

**Authors:** Melania Scarpa, Cesare Ruffolo, Andromachi Kotsafti, Fabio Canal, Francesca Erroi, Silvia Basato, Lucia DallAgnese, Alain Fiorot, Anna Pozza, Paola Brun, Nicol Bassi, Angelo Dei Tos, Carlo Castoro, Ignazio Castagliuolo, Marco Scarpa

**Affiliations:** ^1^Laboratory of Advanced Translational Research, Veneto Institute of Oncology IOV-IRCCS, Padua, Italy; ^2^Clinica Chirurgica I, Azienda Ospedaliera di Padova, Padua, Italy; ^3^Pathology Unit, Treviso Regional Hospital, Treviso, Italy; ^4^Department of Surgery, Oncology and Gastroenterology DISCOG, University of Padua, Padua, Italy; ^5^Department of Molecular Medicine DMM, University of Padua, Padua, Italy; ^6^Oncological Surgery Unit, Veneto Institute of Oncology IOV-IRCCS, Padua, Italy

**Keywords:** colorectal cancer, TLR4, MLH1, mismatch, innate immunity

## Abstract

Patients with mismatch repair (MMR)-deficient colorectal cancer (CRC) have a more favorable prognosis than patients with tumors with intact MMR. In order to obtain further insights on the reasons for this different outcome, we investigated the interplay between MMR genes and TLR4/MyD88 signaling. The cancer genome atlas (TCGA) databases were selected to predict the differential expression of TLR4 in colon cancer and its correlation with MMR genes. Moreover, the expression of MMR genes and TLR4 was evaluated by immunohistochemistry in 113 CRC samples and a cohort of 63 patients was used to assess *TLR4* mRNA expression and *MLH1* epigenetic silencing status. *In vitro*, the effect of *MLH1* knockdown on *TLR4* expression was quantified by Real Time PCR. TLR4 expression resulted dependent on MMR status and directly correlated to MLH1 expression. *In vitro*, *MLH1* silencing decreased *TLR4* expression. These observations may reflect the better prognosis and the chemoresistance of patients with CRC and MMR defects.

## Introduction

Approximately 35% of colorectal cancers occur in the setting of a heritable syndrome, such as hereditary non polyposis colon cancer (HNPCC) syndrome (Barzi et al., 2015). Defects in DNA mismatch repair genes (*MLH1*, *MSH2*, *MSH6*, and *PMS2*) lead to microsatellite instability (MSI-H), which is a hallmark of HNPCC (Ait Ouakrim et al., 2015). However, high-frequency MSI occurs in approximately 15% of sporadic colorectal cancers (CRC) and other tumors (Peltomki, 2001), wherein the mismatch repair genes (MMR) defect develops because of epigenetic inactivation of the *MLH1* gene by DNA methylation (Cunningham et al., 1998; Poynter et al., 2008; Sinicrope et al., 2010). Moreover, miRNAs may also concur to suppress MMR gene expression. In fact, recent studies showed that miR-155 can down-regulate MSH2, MSH6, and MLH1 (Valeri et al., 2010b), whereas miR-21 can down-regulate MSH2 (Valeri et al., 2010a; Luo et al., 2011). Therefore, among the approximately 150,000 new CRC cases diagnosed in the United States in 2008 (Jemal et al., 2008), at least 20,000 patients were expected to have sporadic MMR-deficient tumors (Sinicrope et al., 2010).

Multiple retrospective studies (Halling et al., 1999; Gaf et al., 2000; Lanza et al., 2006; Sinicrope et al., 2006), including a population-based study (Samowitz et al., 2001) and a meta-analysis (Popat et al., 2005), have demonstrated that patients with MMR-deficient colon cancers have a more favorable stage-adjusted prognosis compared with patients whose tumors have intact MMR. However, in spite of the higher mutational load and the consequent higher immune response to cancer cells and high responsiveness toward immune checkpoint blockade, a full explanation for the better outcome of MMR-deficient CRC is still lacking (Ozcan et al., 2018).

Toll-like receptor-4 (TLR4) is the major intracellular signaling complex for bacterial lipopolysaccharides (LPS) (Connolly and ONeill, 2012; Li et al., 2013; Medvedev, 2013) as well as for endogenous proteins such as high mobility group box-1 (HMGB1) (Thada et al., 2013). It induces a signaling cascade that is primarily dependent on Myeloid Differentiation factor 88 (MyD88), a universal adapter protein that leads to the activation of nuclear factor kB (NF-kB) and mitogen-activated protein kinases. The increased expression of TLR4 is a common feature of colorectal adenocarcinoma and TLR4/MyD88 signaling has been associated with poor prognosis (Wang et al., 2010; Beilmann-Lehtonen et al., 2020). Indeed, TLR4 was reported to promote immune escape of human colon cancer cells by inducing immunosuppressive factors as well as apoptosis resistance (Tang and Zhu, 2012). Moreover, mice lacking TLR4 are strongly protected against colon carcinogenesis, substantiating the involvement of TLR4/MyD88 pathway to CRC progression (So and Ouchi, 2010; Tang et al., 2010). Moreover, adenocarcinoma patients with higher TLR4 expression in the stromal compartment had a significantly increased risk of disease progression (Cammarota et al., 2010). The aim of our study was to investigate the interplay between MMR genes and TLR4 expression in colorectal cancer.

## Materials and Methods

### The Cancer Genome Atlas (TCGA) Dataset Analysis

Explorative series consisted of gene expression data from colon adenocarcinoma samples of the TCGA dataset (Cancer Genome Atlas Network, 2012) and TCGA PanCancer Atlas dataset (Liu et al., 2018), which were analyzed through the cBioPortal^1^ (Cerami et al., 2012; Gao et al., 2013).

### Patients

The study was conducted according to the principles of the Declaration of Helsinki and all those participating gave their consent to have their data and anonymized specimens used for scientific purposes.

Immunohistochemical analysis was performed on 113 patients operated on for CRC at the Treviso Regional Hospital and the Ethical Committees for Clinical Experimentation of the Provinces of Treviso and Belluno (study code: VII/RPA-AULSS9) were notified. Inclusion criterion was patients with sporadic colorectal cancers and the exclusion criteria were cancers associated with ulcerative colitis, Crohns disease, or familial adenomatous polyposis. Patients familial and medical histories were retrieved. In particular, presence of positive Bethesda criteria, tumor stage, tumor site were examined. On paraffin-embedded tumor samples from these patients, immunohistochemistry analysis for TLR4 was performed. Mismatch repair genes defects were analyzed evaluating the nuclear expression of MSH2, MLH1, MSH6, and PMS2 on tumor and stromal cells for HNPCC diagnosis.

Moreover, TLR4 gene expression and MLH1 methylation analysis was performed on 63 patients who underwent colonoscopy for screening or postoperative follow up or colonic resection for CRC at the Endoscopical Unit of the Dept of Surgical, Gastroenterological and Oncological Sciences of the University of Padova or at the Surgical Oncology Unit of the Veneto Institute of Oncology from August 2011 to November 2011 (project MICCE1 IOV 2011/53). Characteristics of the two groups of patients are outlined in Table 1.

**TABLE 1:** Patients characteristics.

**Pts used for IHC analysis**			**Cancer**
Subjects			113
Median age (range) years			69 (5480)
Gender (male/female)			59/54
Carcinogenesis stage			Stage I: 11
			Stage II: 31
			Stage III: 47
			Stage IV: 24
Cancer site (%)	Right-sided		59
	Left-sided		44
	Rectum		10

**Pts used for gene expression analysis**	**Healthy controls**	**Adenoma and dysplasia**	**Cancer**

Subjects	30	14	10
Median age (range) years	59.5 (5269) years	61 (5169) years	63 (4974) years
Gender (male/female)	14/16	7/7	7/3
Procedure	Colonoscopy: 30	Colonoscopy: 12	Colonic resection: 8
		RPC: 2	Rectal resection: 2
Carcinogenesis stage	NA	LGD: 9	Stage I: 3
		HGD: 5	Stage II: 2
			Stage III: 4
			Stage IV: 1

*HGD, high grade dysplasia; LGD, low grade dysplasia.*

### Immunohistochemistry and Pathology Assessment

Histology sections (3 m), obtained from formalin fixed, paraffin embedded specimens, were stained with hematoxylin-eosin. Paraffin-embedded tumors (*n* = 89) were analyzed for MLH1, MSH2, MSH6, and PMS2 proteins (Lanza et al., 2006). Immunohistochemical analyses were performed using standard procedures. Primary antibodies used were for MLH1 (clone ES05; Dako, Glostrup, Denmark), PMS2 (clone EP51; Dako), MSH6 (clone EP49; Dako), MSH2 (clone FE11; Dako), TLR4 (clone 76B357.1; Abcam, Cambridge, United Kingdom). Immunocomplexes were detected using an avidin-biotin-peroxidase conjugate and 3-3 di-aminobenzidine tetrahydrochloride chromogen as a substrate (ABC Kit, Vector Laboratories, Burlingame, CA, United States; and DAB kit Dako, Glostrup, Denmark). Slides were scored by a GI expert pathologist (F.C.) as either positive or negative based on the presence or absence of nuclear staining for each MMR protein in the tumor cells. Each slide contained a unique number that enabled blinding with respect to patient identity and clinical characteristics. TLR4 expression was graded on a semi quantitative scale (negative, low, moderate or high). Ten random fields (x63) from each sample were examined.

### Gene Expression Analysis

Total RNA was extracted using the RNeasy Plus Kit (Qiagen) according to the manufacturers protocol. At that point, 0.5 g total RNA was converted to cDNA using the Applied Biosystems cDNA Synthesis kit, again, according to the manufacturers instructions. Specific mRNA transcripts were quantified with SYBR Green PCR Master Mix in an ABI PRISM 7000 Sequence Detection System (Applied Biosystems). The expression of the target molecule was normalized to the expression of the ACTB housekeeping gene. Sequences of PCR primer pairs were for TLR4 fw 5 TTTCCTGCAATGGATCAAGGA 3 rv 5 TTATCTG AAGGTGTTGCACATTCC 3; ACTB fw 5CTGGACTTCGAG CAAGAGAT G3 rv 5AGTTGAAGGTAGTTTCGTGGATG3.

### Methylation Specific PCR

Genomic DNA was extracted using a DNeasy Blood & Tissue Kit (Qiagen) according to the manufacturers directions. Sodium bisulfate modification of gDNA was performed using the EZ DNA Methylation-Gold Kit (Zymo Research) following the manufacturers instructions. Modified DNA was amplified in a total volume of 25 L containing 1 Reaction Buffer, 0.25 mM each dNTP, 0.3 M each primer and 1 U ZymoTaq DNA Polymerase (Zymo Research). The primers for MLH1 methylation-specific PCR were for methylated MLH1 fw 5ACGTAGACGTTTTATTAGGGTCGC3 rv 5CCTCATCG TAACTACCCGCG3 and for unmethylated MLH1 fw 5TTTT GATGTAGATGTTTTATTAGGGTTGT3 rv 5ACCACCTCAT CATAACTACCCACA3. PCR was performed for 45 cycles with annealing temperatures of 56C for 30 s and primer extension at 72C for 60 s using 10 ng bisulfite-modified DNA. The EpiTect PCR Control DNA Set (Qiagen) was used as the positive control for the methylated and unmethylated MLH1 gene. PCR products were resolved by gel electrophoresis and each case was scored as methylated or unmethylated.

### MLH1 Gene Silencing

Caco-2 and SW480 cell lines were purchased from the American Tissue Culture Collection, cultured in DMEM supplemented with 10% FBS and 1X pen/strep solution (all from Life Technologies) and maintained in humidified 37C 5% CO_2_ incubators according to the manufacturers protocol. Specific Silencer Select siRNA for human MLH1 (s224048) and Silencer Select negative control siRNA #1 were purchased from Ambion by Life Technologies. Cells were seeded in 12-well plates and siRNA were transfected when cells reached 50% confluency. For each well, 4 l of Lipofectamine 2000 (Invitrogen by Life Technologies) and 20 pmol of specific or control siRNA were used according to the manufacturers protocol. Silencing efficiency was verified by qRT-PCR 48 h after transfection using the TaqManGene Expression Assay (Applied BioSystems by Life Technologies) for MLH1 (HS00179866_m1).

### Flow Cytometry

Cell lines treated with siRNAs for 48 h were trypsinized and washed with 1X PBS before staining. For staining, 10^5^ cells were suspended in PBS/2% FBS with anti-human TLR4-PECy7 (eBioscience-Thermo Fisher Scientific) antibody for 30 min on ice. Flow cytometric analysis was performed using a FACSCalibur based on CellQuest software (BD-Becton Dickinson, Franklin Lakes, United States).

### Statistical Analysis

Statistical analysis was carried out with STATISTICA 5.1 software. The results are presented as mean +/SEM unless otherwise specified. Non parametric MannWhitney *U* test for independent variables or Kruskall-Wallis ANOVA for multiple variables were used for comparison as appropriate. The Fisher exact test was used to compare the frequency of patients expressing TLR4 at immunohistochemistry. Kaplan-Meier curves were designed to assess patients overall survival and log-rank test was used to compare groups according to the expression of TLR4. Differences were considered significant at *p* < 0.05.

## Results

### Patients With MMR Genes Deficient Colon Cancer Exhibit TLR4 Downregulation

**FIGURE 1:**
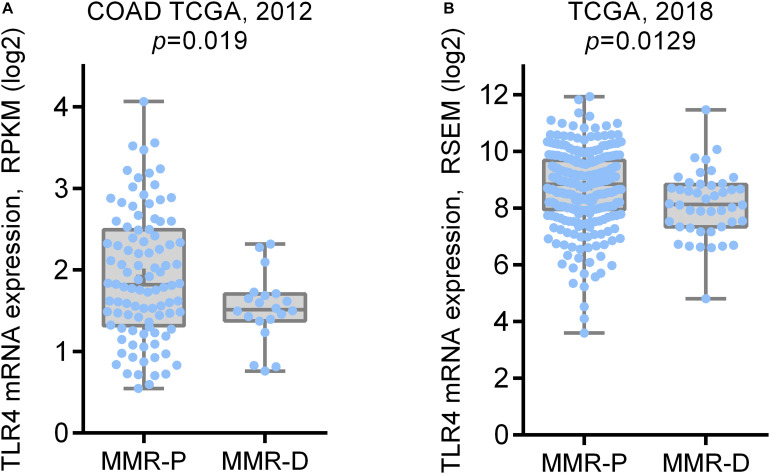
TLR4 mRNA expression in colon adenocarcinoma TCGA dataset according to MMR status. **(A)** TLR4 mRNA expression in MMR proficient (MMR-P: MSS subtype) and MMR deficient (MMR-D: MSI-H and MSI-L subtype) samples in COAD TCGA dataset; **(B)** TLR4 mRNA expression in MMR proficient (CIN, GS, and POLE subtypes) and MMR deficient (MSI subtype) samples in TCGA PanCancer Atlas dataset.

TCGA COAD dataset consisted of 172 samples, including 23 MSI-H (13.4%), 33 MSI-L (19.2%), and 116 MSS (67.4%) tumor samples. TCGA PanCancer Atlas dataset comprised 378 samples of colon adenocarcinoma, including 44 MSI (11.6%), 206 CIN (54.5%), 36 GS (9.5%), and 5 POLE mutations (1.3%). We compared the mRNA expression levels of TLR4 in MSI tumors (MMR genes deficient, MMR-D) vs. non-MSI tumors (MMR genes proficient, MMR-P). In both datasets, TLR4 mRNA level was significantly downregulated in the MMR-D group compared to the MMR-P group (Figure 1A *p* = 0.019, and Figure 1B *p* = 0.0129). These results suggest that MMR genes deficiency in colon adenocarcinoma is associated to decreased TLR4 expression.

### Expression of TLR4 in a Tissue Microarray of Colon Cancer According to MMR Status

To validate the observation made in TCGA databases, TLR4 protein expression was retrospectively analyzed in a group of 113 consecutive patients who had colonic resection for CRC. The mean age of these cases was 69 years (range 5480, 59 men, and 54 women). Patients with stage I or II were 42 and those with stage III or IV were 71. In 59 patients, CRC was located in the right-transverse colon, in 44 in the left and sigmoid colon and in 10 in the rectum. In this group, 48 (42.5%) patients presented at least one Bethesda criteria.

**FIGURE 2:**
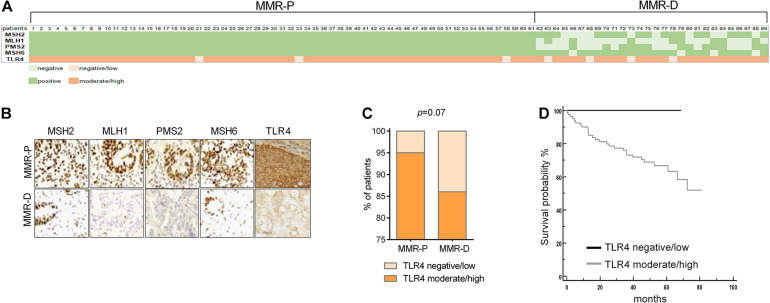
Expression of TLR4 in a tissue microarray of colon cancer according to MMR status. **(A)** Heat map showing MMR genes expression (light green = no expression; dark green = expression). TLR4 expression was graded as low (no or mild expression = light orange) or high (moderate or high expression = dark orange). **(B)** Representative images of MMR genes and TLR4 expression according to the presence of MMR deficiency at immunohistochemistry: **(C)** data expressed as frequency of patients with low or high expression. **(D)** Kaplan-Meier estimate was used to perform the survival analysis and the log-rank test was used to compare groups according to TLR4 expression.

In our series, 28 (24.7%) patients had at least one MMR gene deficiency in CRC tissue at immunohistochemical analysis (Figures 2A,B). MLH1 was deficient in 20, MSH2 in 10, PMS2 in 15 patients, and MSH6 was deficient in 4 patients. Four patients had a synchronous deficiency of 3 MMR genes and 12 presented with deficiency of 2 MMR genes. Notably, patients with MMR gene deficiency exhibited a trending decrease of TLR4 protein expression (*p* = 0.07) (Figure 2C). No direct influence of MMR deficiency on survival was observed. Low TLR4 expression seemed associated to a better overall survival but the difference did not resulted statistically significant (*p* = 0.18) (Figure 2D).

### Correlation of TLR4 With MLH1 Expression

**FIGURE 3:**
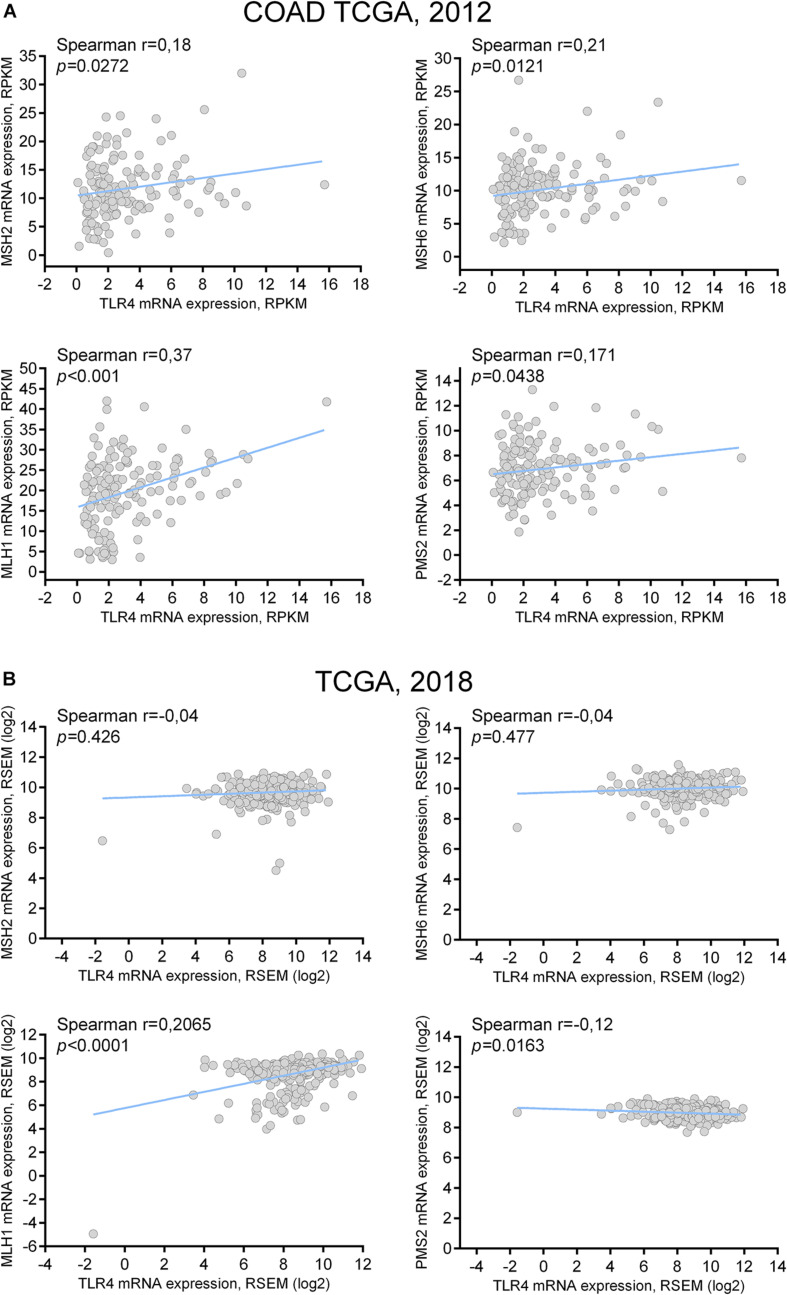
TLR4 mRNA coexpression analysis with MMR genes: correlation with MLH1. **(A)** TLR4 mRNA coexpression analysis with MSH2, MSH6, MLH1, and PMS2 mRNA in tumor samples of COAD TCGA dataset. **(B)** TLR4 mRNA coexpression analysis with MSH2, MSH6, MLH1, and PMS2 mRNA in tumor samples of TCGA PanCancer Atlas dataset.

The analyses on the TCGA databases were next performed to verify the correlation of TLR4 mRNA expression with single MMR genes expression. There were significant positive correlations between TLR4 and MMR genes (MSH2, MSH6, MLH1, and PMS2) in TCGA COAD dataset (Figure 3A), but MSH2, MSH6, and PMS2 positive correlations were not confirmed in TCGA PanCancer Atlas colon adenocarcinoma dataset (Figure 3B). However, MLH1 gene expression resulted significantly correlated with TLR4 expression in both datasets.

### Reduced Expression of TLR4 in MLH1-Deficient Colon Tissue and Cells

**FIGURE 4:**
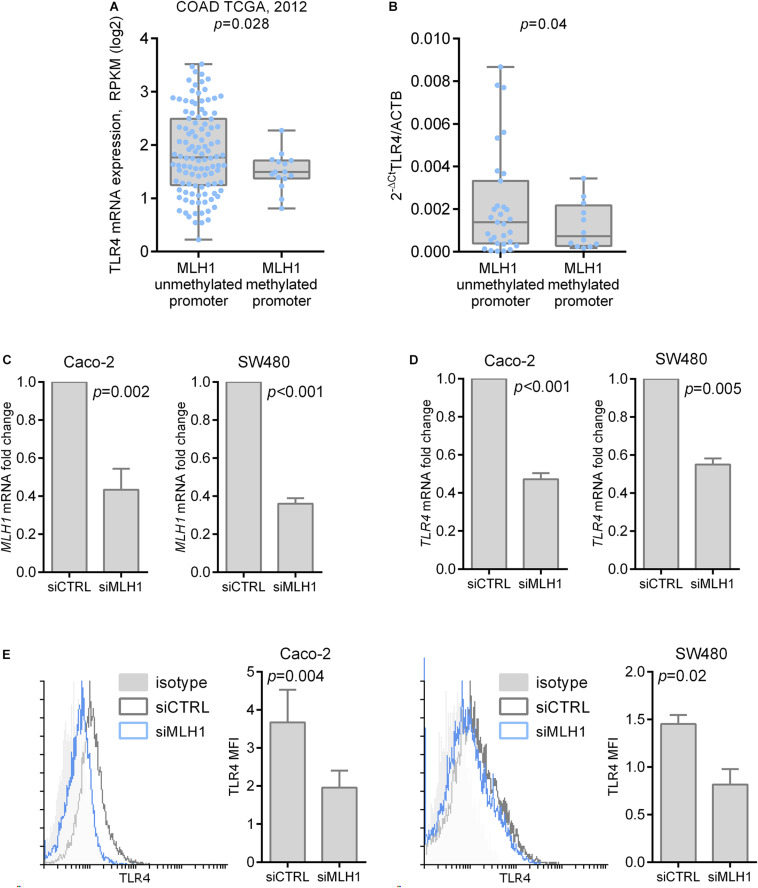
TLR4 expression is influenced by MLH1 deficiency. **(A)** TLR4 mRNA expression according to epigenetic silencing of MLH1 in tumor samples of COAD TCGA dataset. **(B)** TLR4 mRNA expression according to epigenetic silencing of MLH1 in the healthy colon mucosa of 63 patients. **(C)** Efficiency of MLH1 specific silencing in Caco-2 and SW480 cells quantified by qRT-PCR. **(D)** TLR4 mRNA expression quantification in Caco-2 and SW480 cells transfected with specific MLH1 siRNA or control siRNA. **(E)** Cell surface expression of TLR4 in Caco-2 and SW480 cells transfected with specific MLH1 siRNA or control siRNA was measured by flow cytometry.

Next, we explored whether MLH1 deficiency could affect TLR4 expression. Data about the epigenetic silencing status of MLH1 were available for 138 samples in the TCGA COAD dataset, thus we analyzed TLR4 expression according to MLH1 epigenetic status. As shown in Figure 4A, TLR4 mRNA expression resulted significantly decreased in tumor samples characterized by MLH1 epigenetic silencing (*p* = 0.0284). To further corroborate this observation, we assessed the expression of TLR4 mRNA and the promoter methylation status of MLH1 in the healthy colonic mucosa of 63 patients who had colonoscopy for cancer screening or cancer follow up or who had colonic resection for colorectal cancer. As shown in Figure 4B, TLR4 mRNA expression was significantly lower in healthy mucosa specimen with MLH1 promoter methylation compared to samples with unmethylated MLH1 promoter (*p* = 0.04). The results obtained suggest that MLH1 deficiency alters TLR4 mRNA expression both in normal and tumor colonic mucosa. Moreover, we observed a significant reduction in TLR4 mRNA and protein expression upon specific silencing of MLH1 in colon epithelial cell line Caco-2 and SW480 (Figures 4CE). Taken together, our data suggest that MLH1 deficiency affect TLR4 expression in colon epithelial cells.

## Discussion

Patients with MMR-deficient CRC have a more favorable stage-adjusted prognosis compared with patients whose tumors have intact MMR; however, the reasons for this better outcome are still not univocal. In this study we investigated for the first time the relationship between MMR genes deficiency and innate immunity in CRC, and provided evidence for a direct role of MLH1 in the regulation of TLR4 expression.

Some recent reports showed that MMR gene deficient tumors were selectively characterized by a highly upregulated active Th1/CTL microenvironment (Llosa et al., 2015; Scarpa et al., 2015), pointing to a role of adaptive immunity for the improved prognosis of these patients (Galon et al., 2006). Moreover, MLH1 inactivation was proven to cause an hypermutation status that increases tumor neoantigens, which in turn trigger long-lasting immune surveillance (Germano et al., 2017). Because an increased expression of TLR4 in CRC is common and TLR4/MyD88 signaling is associated with poor prognosis (Wang et al., 2010; Kantola et al., 2012; Tye and Jenkins, 2013), we analyzed TLR4 expression in colorectal adenocarcinoma specimen according to MMR status. The main findings of our study was that TLR4 expression is dependent on MMR status and it was directly correlated to MLH1 expression. Moreover, MLH1 silencing *in vitro* and *in vivo* by epigenetic silencing resulted in TLR4 decreased expression, thus demonstrating that MLH1 contributes to TLR4 expression regulation.

Over-activation of TLR4 in intestinal epithelial cells (IEC) promotes the recruitment of macrophages and leukocytes to the lamina propria and subsequently results in IL-6- mediated STAT3 activation to facilitate the production of COX2/PGE2 that encourages IEC proliferation and survival in CRC (Tye and Jenkins, 2013). Moreover, the binding of the HMGB1, a TLR4 endogenous ligand, may activate NF-kB, promoting gene transcription leading to subsequent activation of downstream factors, including mitogen-associated protein kinase (MAPK) and interferon (IFN) regulatory factors (Apetoh et al., 2007; Davoodi et al., 2013).

Therefore, the observation that in CRC cells and in colonic mucosa MLH1 deficiency leads to TLR4 expression decrease may give a further explanation of the better prognosis of patients with a MSI-H CRC. Moreover, MSI-H CRC are notoriously resistant to 5-fluoruracil (5-FU) chemotherapy. 5-FU was demonstrated to activate the Poly (ADP-ribose) polymerase (PARP) that can stimulate the release of HMGB1 from its association with chromatin (Davoodi et al., 2013). Chemotherapy-induced cell death triggers the release of the high-mobility group box 1 protein (HMGB1), which stimulates TLR4 and elicits an immune response that is essential for the success of the therapy (Apetoh et al., 2007). The down-regulation of TLR4 in patients with MMR gene deficient CRC may add a further explanation of chemoresistance of MSI-H CRC besides the effect of miRNA-21 on MSH2 (Valeri et al., 2010a).

In conclusion, our study showed that in CRC patients low TLR4 expression tended to be more frequent in patients with MMR gene deficiency and that TLR4 mRNA expression was significantly decreased when MLH1 was epigenetically silenced. Our *in vitro* experiments proved for the first time that MLH1 influences TLR4 expression. These data provide further explanation of the better prognosis and of the chemo-resistance of patients with an MSI-H CRC.

## Data Availability Statement

Publicly available datasets were analyzed in this study. This data can be found here: TCGA.

## Ethics Statement

The studies involving human participants were reviewed and approved by the Treviso Regional Hospital and the Ethical Committees for Clinical Experimentation of the Provinces of Treviso and Belluno (study code: VII/RPA-AULSS9). The patients/participants provided their written informed consent to participate in this study.

## Author Contributions

MaS conceived the study, participated in its design and coordination, and drafted the manuscript. CR participated in the design of the study and in the drafting of the manuscript. MeS performed the gene expression analysis, the methylation specific PCR, the MLH1 gene silencing, and drafted the manuscript. FC and AK carried out the immunoassays. FE, SB, and PB collected the tissues samples. LD and AF retrieved patients history and revised patients records. AP performed the statistical analysis. NB, AD, IC, and CC participated in the design of the study and in the critical revision of the draft. All authors read and approved the final manuscript.

## Conflict of Interest

The authors declare that the research was conducted in the absence of any commercial or financial relationships that could be construed as a potential conflict of interest.

## Footnotes

1 www.cbioportal.org
